# A high-throughput drug screening identifies luteolin as a therapeutic candidate for pathological cardiac hypertrophy and heart failure

**DOI:** 10.3389/fcvm.2023.1130635

**Published:** 2023-03-14

**Authors:** Zhenya Wang, Wei Shi, Taibo Wu, Tian Peng, Xiaoming Wang, Shuaiyang Liu, Zifeng Yang, Jia Wang, Peng-Long Li, Ruifeng Tian, Ying Hong, Hailong Yang, Lan Bai, Yufeng Hu, Xu Cheng, Hongliang Li, Xiao-Jing Zhang, Zhi-Gang She

**Affiliations:** ^1^ Department of Cardiology, Renmin Hospital, School of Basic Medical Science, Wuhan University, Wuhan, China; ^2^ Institute of Model Animal, Wuhan University, Wuhan, China; ^3^ Gannan Innovation and Translational Medicine Research Institute, Key Laboratory of Prevention and Treatment of Cardiovascular and Cerebrovascular Diseases, Ministry of Education, Gannan Medical University, Ganzhou, China; ^4^ Medical Science Research Center, Zhongnan Hospital of Wuhan University, Wuhan, China

**Keywords:** luteolin, cardiac hypertrophy, heart failure, peroxisome proliferator activated receptor γ, fatty acid metabolism, glucose metabolism

## Abstract

**Background:**

Pathological cardiac hypertrophy is commonly resulted from sustained pressure overload and/or metabolic disorder and eventually leads to heart failure, lacking specific drugs in clinic. Here, we aimed to identify promising anti-hypertrophic drug(s) for heart failure and related metabolic disorders by using a luciferase reporter-based high-throughput screening.

**Methods:**

A screen of the FDA-approved compounds based on luciferase reporter was performed, with identified luteolin as a promising anti-hypertrophic drug. We systematically examined the therapeutic efficacy of luteolin on cardiac hypertrophy and heart failure *in vitro* and *in vivo* models. Transcriptome examination was performed to probe the molecular mechanisms of luteolin.

**Results:**

Among 2,570 compounds in the library, luteolin emerged as the most robust candidate against cardiomyocyte hypertrophy. Luteolin dose-dependently blocked phenylephrine-induced cardiomyocyte hypertrophy and showed extensive cardioprotective roles in cardiomyocytes as evidenced by transcriptomics. More importantly, gastric administration of luteolin effectively ameliorated pathological cardiac hypertrophy, fibrosis, metabolic disorder, and heart failure in mice. Cross analysis of large-scale transcriptomics and drug-target interacting investigations indicated that peroxisome proliferator activated receptor γ (PPARγ) was the direct target of luteolin in the setting of pathological cardiac hypertrophy and metabolic disorders. Luteolin can directly interact with PPARγ to inhibit its ubiquitination and subsequent proteasomal degradation. Furthermore, PPARγ inhibitor and PPARγ knockdown both prevented the protective effect of luteolin against phenylephrine-induced cardiomyocyte hypertrophy *in vitro*.

**Conclusion:**

Our data clearly supported that luteolin is a promising therapeutic compound for pathological cardiac hypertrophy and heart failure by directly targeting ubiquitin-proteasomal degradation of PPARγ and the related metabolic homeostasis.

## Introduction

Heart failure (HF) causes a serious social and economic burden, with a prevalence of approximately 1%–2% (1). Cardiac hypertrophy caused by hemodynamic overload is a critical irritation in heart failure (2). The pathogenesis of cardiac hypertrophy involves alterations in cardiac myocyte metabolism, oxidative stress, endoplasmic reticulum stress and autophagy (3), as well as alterations in fibroblasts, inflammatory cells, and endothelial cells (4). A group of drugs, including angiotensin converting enzyme inhibitors, β-adrenergic receptor blockers, and angiotensin receptor blockers, exhibit clinical benefit in inhibiting the progression of cardiac hypertrophy and HF (5). However, decades of clinical application of these drugs failed to reduce the absolute number of HF patients mainly due to the sustained exacerbation of risk factors including obesity, diabetes, and other causal factors, as well as an ageing population (6). Therefore, it is urgently necessary to gain insight into the pathogenesis of cardiac hypertrophy and HF and identify new therapeutic approaches.

De novo drug development generally requires a significant investment of time, manpower and costs and the success rate still needs to be improved. An alternative strategy to reduce the duration and costs of drug development is to explore new indications for existing drugs, which can take advantage of the pre-existing pharmacodynamic/pharmacokinetic and toxicology properties of many drugs approved for human use. For example, raloxifene, which is approved for the treatment of osteoporosis, has been found to be beneficial in the treatment of breast cancer in recent studies (7).

Here, we applied luciferase reporter-based high-throughput screening on FDA-approved chemical compounds library (include 2,570 compounds) to identify potential anti-hypertrophic drugs for heart failure and the related metabolic disorders. Among several positive hits, luteolin emerged as the most robust candidate against cardiomyocyte hypertrophy. Luteolin, one of the most prevalent flavones, possesses anti-oxidative, anti-tumor, anti-apoptotic, and anti-inflammatory properties (8–10). Although its potential protective effects on cardiomyocyte hypertrophy and fibrosis have also been proposed (11–14), it is remains to be verified regarding whether luteolin has a sustained protective effect throughout the progression of pressure overload-induced pathological cardiac hypertrophy and HF. Furthermore, the panoramic effects of luteolin in the heart and the specific regulatory mechanism underlying its effects are also unclear. All these information is fundamentally essential for repurposing luteolin as a potential candidate for pathological cardiac hypertrophy and HF.

Here, we successively demonstrated that luteolin blocked phenylephrine-induced cardiomyocyte hypertrophy and ameliorated pressure overload-induced cardiac hypertrophy, fibrosis, metabolic disorder, and HF in mice. Mechanistically, we have demonstrated that peroxisome proliferator activated receptor γ (PPARγ) is the direct target of luteolin in the setting of pathological cardiac hypertrophy and the related metabolic disorders. Luteolin can directly interact with PPARγ to inhibit its ubiquitination and subsequent proteasomal degradation. In summary, we here found out from FDA-approved drug library that luteolin emerged as a therapeutic candidate for pathological cardiac hypertrophy and heart failure by directly suppressing ubiquitin-proteasomal degradation of PPARγ and metabolic homeostasis.

## Methods

### Animals

All mice were placed in an environment with controlled light cycles, temperature, and humidity. The cardiac hypertrophy model was constructed by transverse aortic constriction (TAC) surgery as previously described (15). Briefly, male mice with C57BL/6 background (10-week-old; weight: 25–27 g) were anesthetized *via* i.p. injection of pentobarbital sodium (90 mg/kg, P3761, Sigma-Aldrich). After exposing the transverse aorta, the transverse aorta was ligated transversely with 7–0 silk and a 26-gauge needle. Identical operation without ligation the transverse aorta was performed in the sham operation group.

Seven days after TAC or sham surgery, mice were randomly given vehicle or luteolin treatment. Mice were administered orally with luteolin (40 mg/kg) or vehicle [1% carboxymethyl cellulose sodium (CMC-Na)] daily for 11 consecutive weeks. After 4, 8 and 12 weeks of TAC or sham surgery, heart function was respectively measured by echocardiography as previously described (16). At the end of the experiment, the mice were anesthetized using the above method and then subjected into euthanasia *via* cervical dislocation, and the heart, lung, and tibia were collected for further examinations.

### Echocardiography

Echocardiography was used to evaluate mice cardiac function as described previously (16). A small animal ultrasound imaging system (Mylab30CV, ESAOTE, S. P. A) was used to perform echocardiography. The left ventricle was evaluated on both long- and short-axis views of the parasternal sternum as described previously. The echocardiography operator is not informed about the grouping of mice.

### Histological analysis

Twelve weeks after TAC or sham surgery, mice hearts were collected. Hearts were macerated in 10% formalin and subsequently encapsulated in paraffin. Paraffin-embedded mice hearts were sectioned transversely (5 μm). Subsequently, hematoxylin-eosin and picrosirius red staining (for collagen volume fraction analysis) were executed. These analyses were performed using Image-Pro Plus 6.0. At least one hundred cardiomyocytes were examined in each section, and the collagen volume fraction was computed as picrosirius red staining area divided by total area.

### Primary cardiomyocytes isolation, cell treatment, and immunofluorescence staining

Primary cardiomyocytes were obtained from Sprague-Dawley rat (1–2 days) hearts in accordance with previously described (17). Primary neonatal rat cardiomyocytes (NRCMs) were incubated in the DMEM/F12 medium with 10% fetal bovine serum, 1% penicillin/streptomycin, and 0.2 mM BrdU for 48 h. After culturing NRCMs in serum-free DMEM/F12 for 12 h, hypertrophy was induced by adding 50 μM phenylephrine (PE, P6126, Sigma) for 24 h. DMEM medium with 1% penicillin/streptomycin and 10% fetal bovine serum was used for the incubation of H9C2 cells and HEK 293 T cells.

The size of the cardiomyocyte surface was assessed by immunofluorescence staining for α-actinin after 24 h of incubation with PBS or PE as previously described in the established protocol (15, 16). Briefly, cardiomyocytes were successively soaked in 4% formaldehyde and 0.2% Triton-X 100 (T8787, Sigma-Aldrich). Then the cells were stained with α-actinin (1:100 dilutions, A7811, Sigma) and appropriate secondary antibody (1:200 dilutions, A11061, Invitrogen). The size of the cardiomyocyte surface was examined with Image-Pro Plus 6.0.

### Plasmid and lentivirus construction

Rat *Bnp* (b-type natriuretic peptide) promoter (−2147, +132 bp) and *Myh7* (myosin heavy chain 7) promoter (−2500, +89 bp) were expanded and cloned into pGL3-promoter luciferase reporter vector to obtain promoter reporter plasmids. The lentiviral plasmids encoding sh*RNA* for *Pparγ* was constructed in the pLKO.1 vector. HEK 293 T cells were transfected with pLKO-sh*RNA* or pLKO-sh *Pparγ* along with the packaging vectors pSPAX2 and pMD2G. After incubation of cells at 37°C for 40 h, lentiviral suspensions were harvested for infection with H9C2 cells followed by puromycin selection for one week for various analyses. Primers for plasmid and lentivirus construction are provided in Supplementary Table S1.

### FDA-approved library screening

HEK 293 T cells were co-transfected with a plasmid expressing Firefly luciferase and a plasmid expressing Renilla luciferase. After 24 h, FDA-approved compounds (20 μM, L1300, Selleckchem), containing 2,570 compounds, were added separately to the culture medium. The cells were lysed using lysis solution, and the luciferase activity was examined by adding luciferase assay substrate (E1980, Promega). The results were shown as log2-fold change normalized to control.

### Cell viability assay

The Cell Counting Kit (CCK-8, Beyotime) was applied to assay the cell viability of NRCMs. NRCMs were incubated in 96-well plates with 2 × 10^4^ cells per well and subsequently treated with different concentrations of luteolin. After the addition of CCK-8 solution, the NRCMs were further incubated. After 24 h of incubation, the absorbance of each well at 450 nm was detected.

### Immunoprecipitation assays

After 24 h transfection with the appropriate plasmids, HEK293T cells were treated with biotin-linked luteolin (20 μM) or biotin for another 4 h and then lysed in pre-cooled immunoprecipitation (IP) buffer. Add the specified antibodies and protein G Bestarose 4FF beads (AA304307, Bestchrom) to the lysate supernatant and incubate for 4 h. The immunocomplexes were washed with pre-cooled IP buffer and subsequently collected for western blot analysis.

### Western blot

Proteins were obtained from cardiac tissue and cardiomyocytes. The BCA Protein Assay Kit (23225, Thermo) was applied to detect protein levels. Proteins were isolated by SDS-PAGE and electrotransferred to PVDF membranes, which were successively incubated with primary and secondary antibodies. Protein signals were detected using the ChemiDoc MP Imaging System (Bio-Rad). GAPDH served as the loading control. All antibodies are shown in Supplementary Table S2.

### Ubiquitination assays

Cultured HEK 293 T cells were collected and lysed in SDS lysis buffer including protease inhibitor cocktail (04693132001, Roche). The lysates were centrifuged (12,000 rpm for 15 min), and the supernatants were analyzed by immunoprecipitation assays with indicated antibodies, followed by western blot analyses.

### Quantitative real-time PCR

RNA was derived from mice heart tissue and NRCMs with Trizol regent (T9424, Sigma-Aldrich), and reverse transcription was conducted by the HiScript III RT SuperMix for qPCR (R323-01, Vazyme). Quantitative real-time PCR was conducted to detect gene expression level using SYBR Green (Q311-03, Vazyme), and *Gapdh* served as internal reference gene (18). The real-time PCR primers are shown in Supplementary Table S3.

### RNA-sequencing and data processing

RNA extracted from mice hearts and cultured NRCMs was used for library preparation. Single-end RNA-seq was carried out with MGISEQ-2000 (MGI, China). Based on the Ensembl mouse (mm10/GRCm38) genome, gene sequence alignment on clean reads was conducted by HISAT2 software (version 2.1.0). SAMtools software (version 1.4.1) was used to transform the mapped fragments into the Binary Alignment Map files. Then, the reads count value of the genes were computed using StringTie (version v1.3.3b). DESeq2 (v1.2.10) software was used for differentially expressed genes identification by processing read count information. Gene with adjusted *P*-values less than 0.05 and a fold change larger than 1.5 was recognized as differentially expressed gene. Gene Ontology analysis, Gene Set Enrichment Analysis (GSEA), and Kyoto Encyclopedia of Genes and Genomes analysis were conducted by R package clusterProfiler (version 4.2.2). Gene sets with adjusted *P* value of less than 0.05 were recognized as statistically significant. The R package gmodels (version 2.18.1) was used for principal component analysis.

### Data analysis

SPSS 22.0 was applied to analyze all results and the results were presented as the mean ± SD. When the data were normally distributed, 2 group comparisons were conducted by 2-tailed Student t test, and multiple comparisons were conducted by one-way ANOVA. When data were skewed distributed, multiple comparisons were conducted by Kruskal-Wallis test. *P *< 0.05 was regarded as statistically significant.

## Results

### Luteolin emerged as a therapeutic candidate for pathological cardiac hypertrophy in the FDA-approved chemical compounds screen

**Figure 1:**
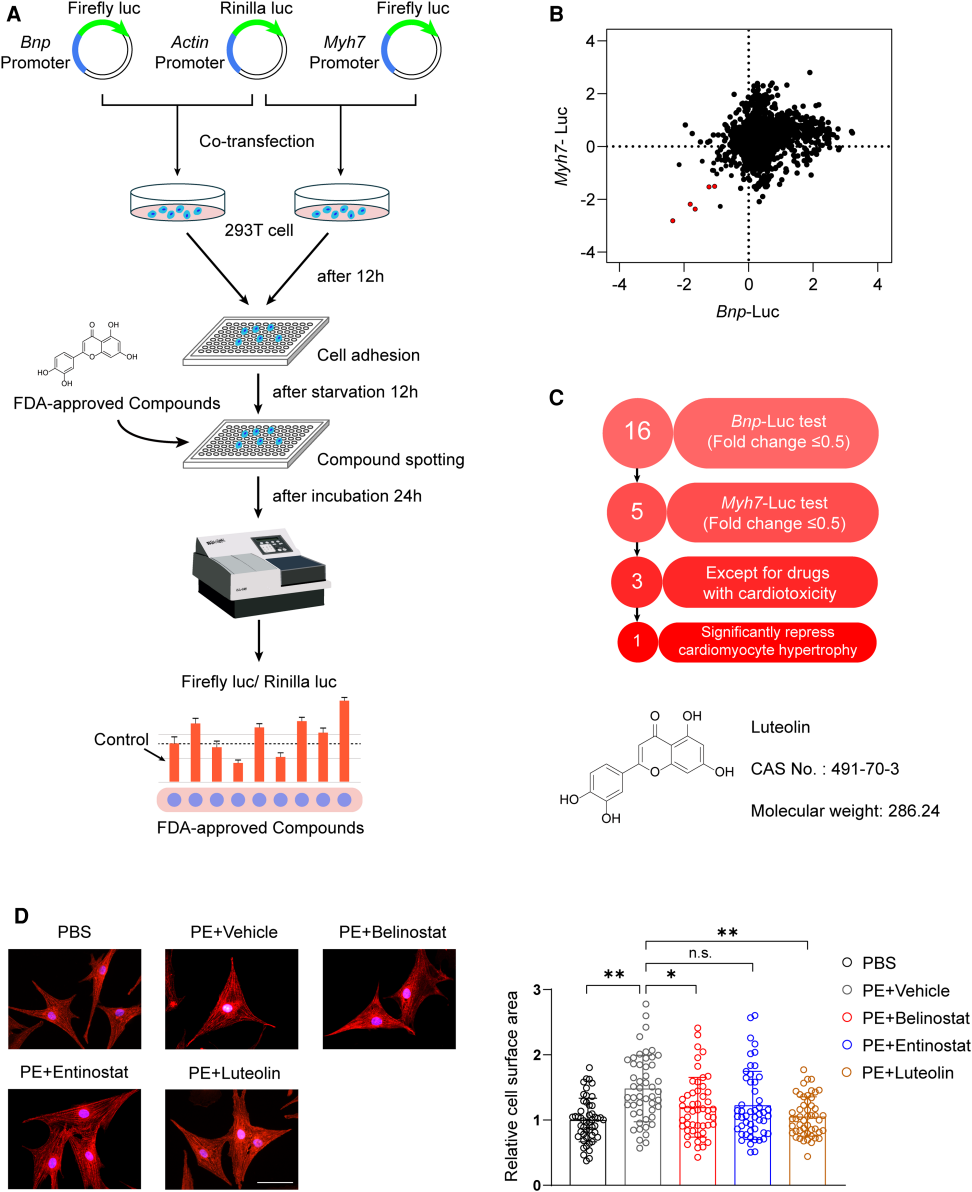
Luteolin emerged as a therapeutic candidate for pathological cardiac hypertrophy in the FDA-approved chemical compounds screen. (**A**) Schematic illustration of the experimental workflow of the luciferase-based FDA-approved chemical compounds screen. (**B**) The scatter plot demonstrating *Myh7* and *Bnp* luciferase activity in HEK 293 T cells treated with FDA-approved compounds. Red dots represent each FDA drug that inhibited both *Myh7* and *Bnp* luciferase reporter activities for more than 50%. (**C**) The advancement criterion for screening out the most effective compound on *Myh7* and *Bnp* luciferase activity downregulation and cardiomyocyte hypertrophy inhibition. (**D**) Representative immunofluorescence images (left) of α-actinin staining and quantitative results of the cell surface area (right) of NRCMs treated with PBS, PE (50 μM), PE + entinostat, PE + belinostat, or PE + luteolin for 24 h (*n* ≥ 50 cells per group). Scale bar, 50 μm. The data shown are representative of three independent experiments. Values are presented as mean ± SD. *P < 0.05, **P < 0.01, n.s., no significant difference. FDA, the United States Food and Drug Administration; NRCMs, Primary neonatal rat cardiomyocytes; HEK 293 T cells, human embryonic kidney 293 T cells; luc, luciferase; *Bnp*, b-type natriuretic peptide; *Myh7,* myosin heavy chain 7; PE, phenylephrine.

Upregulation of *Myh7* and *Bnp* transcriptional activity is an indication of the risk for developing cardiac hypertrophy (19, 20). To explore potential therapeutic drugs for cardiac hypertrophy and HF, our study screened the effects of FDA-approved chemical compounds library on the transcriptional activity of *Myh7* and *Bnp* based on luciferase reporter assays (Figure 1A). The compounds were added to HEK 293 T cells transfected with *Myh7* and *Bnp* promoter reporter plasmids, respectively, and luciferase activity was assayed after 24 h of incubation. Among 2,570 compounds, a total of five were screened as the candidates according to the criteria of downregulating *Myh7* and *Bnp* promoter activity for more than 50% (Figure 1B). After excluding two candidate compounds with significant cardiotoxicity, three candidate compounds were further examined for their effects on PE-induced cardiomyocyte hypertrophy (Figure 1C). Notably, luteolin, a flavonoid from a variety of plants (10), produced the most pronounced protective effect compared to dimethyl sulfoxide (DMSO) as indicated by immunofluorescence staining (Figures 1C,D).

### Luteolin ameliorates PE-induced cardiomyocyte hypertrophy in primary cardiomyocytes

**Figure 2:**
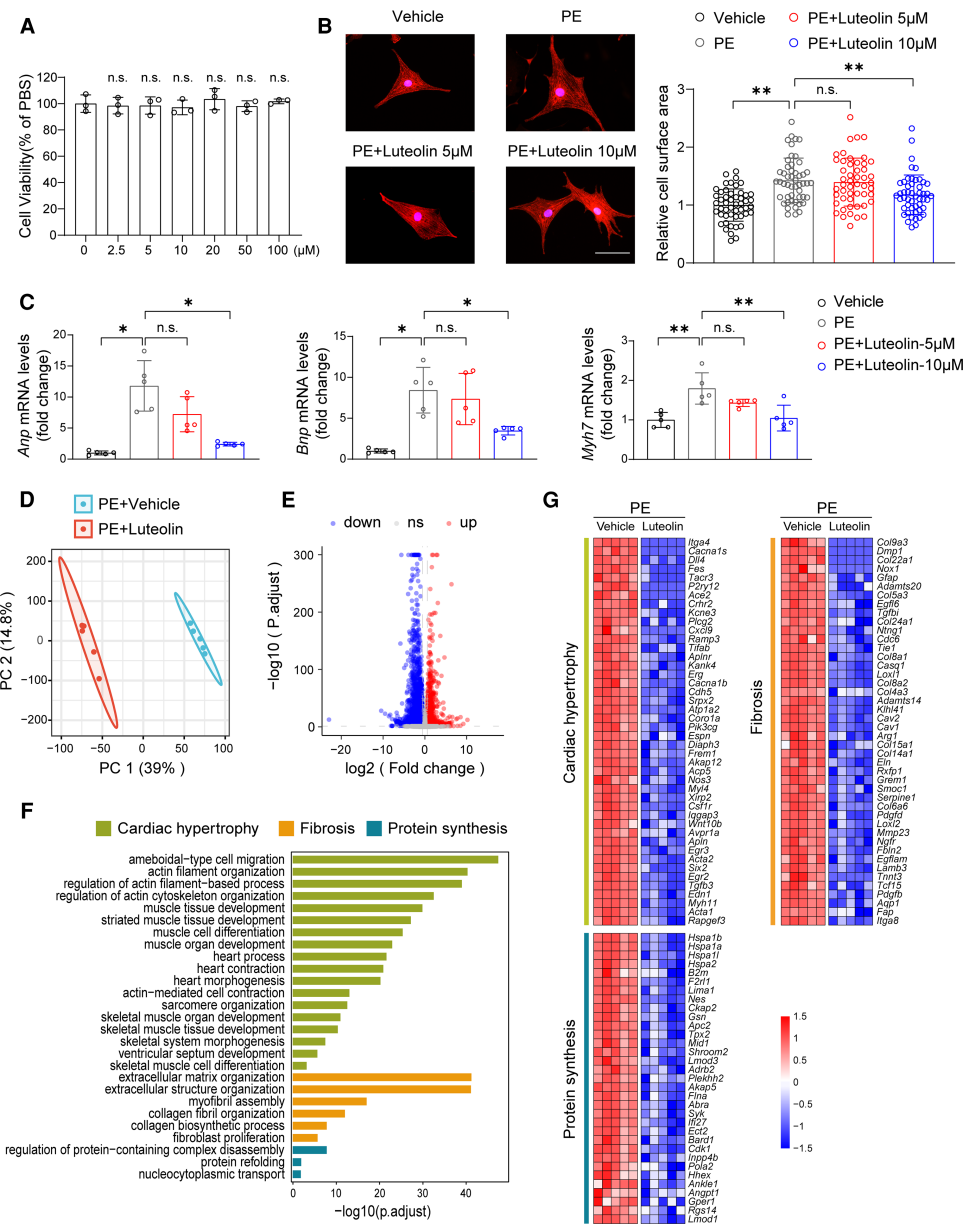
Luteolin ameliorates PE-induced cardiomyocyte hypertrophy in primary cardiomyocytes. (**A**) Relative cell viability of NRCMs after treatment with different concentrations of luteolin. The data shown are representative of three independent experiments. n.s., no significant difference compared to the 0 μM group. (**B**) Representative immunofluorescence images (left) of α-actinin staining and quantitative results of the cell surface area (right) of NRCMs treated with PBS, PE (50 μM), or PE + luteolin (5 or 10 μM) for 24 h (*n* ≥ 50 cells per group). Scale bar, 50 μm. The data shown are representative of three independent experiments. (**C**) Relative mRNA levels of cardiac hypertrophy marker genes (*Anp*, *Bnp*, and *Myh7*) in NRCMs treated with PBS, PE (50 μM), or PE + luteolin (5 or 10 μM) for 24 h (*n* = 5 independent experiments). (**D**) Principal component analysis showing the global sample distribution profiles between groups based on the RNA-sequencing data. (**E**) Volcano plot analysis showing a huge number of differentially expressed genes between the two groups. Genes with adjusted *P*-values less than 0.05 and a fold change larger than 1.5 was recognized as differentially expressed genes. (**F**) Gene set enrichment analysis of molecular events involved in cardiac hypertrophy, fibrosis, and protein synthesis in RNA-sequencing data. (**G**) Heatmap showing the significantly altered genes related to cardiac hypertrophy. Values are presented as mean ± SD. *P < 0.05, **P < 0.01, n.s., no significant difference. PE, phenylephrine; *Anp*, atrial natriuretic peptide; *Bnp*, b-type natriuretic peptide; *Myh7,* myosin heavy chain 7.

The cellular safety of luteolin was confirmed by its non-significant impact on cell viability of NRCMs (Figure 2A). We continued to treat PE-stimulated NRCMs with different dosages of luteolin to further evaluate its effect on the hypertrophy of NRCMs. Immunostaining results demonstrated that luteolin inhibited the increase in surface area of cardiomyocytes in a dose-dependent way (Figure 2B). Similarly, the mRNA levels of *Anp* (atrial natriuretic peptide), *Bnp*, and *Myh7* were also significantly inhibited by luteolin (10 μM) (Figure 2C). To explore the effect of luteolin at the panoramic molecular level, RNA-sequencing analysis was conducted on PE-treated NRCMs in the presence or absence of luteolin. By principal component analysis, the transcriptome profiles were clearly divided into two clusters (Figure 2D). The volcano plot results indicated a huge number of differentially expressed genes between the two groups (Figure 2E). GSEA analysis based on gene ontology database showed that the genes modulated by luteolin were mainly concentrated in cardiac hypertrophy, fibrosis, and protein synthesis (Figure 2F). The heatmap showed that luteolin markedly repressed the expression levels of genes associated with the aforesaid pathways (Figure 2G). These results show that luteolin inhibits cardiomyocyte enlargement *in vitro*.

### Luteolin inhibits cardiac dysfunction induced by pressure overload in mice

**Figure 3:**
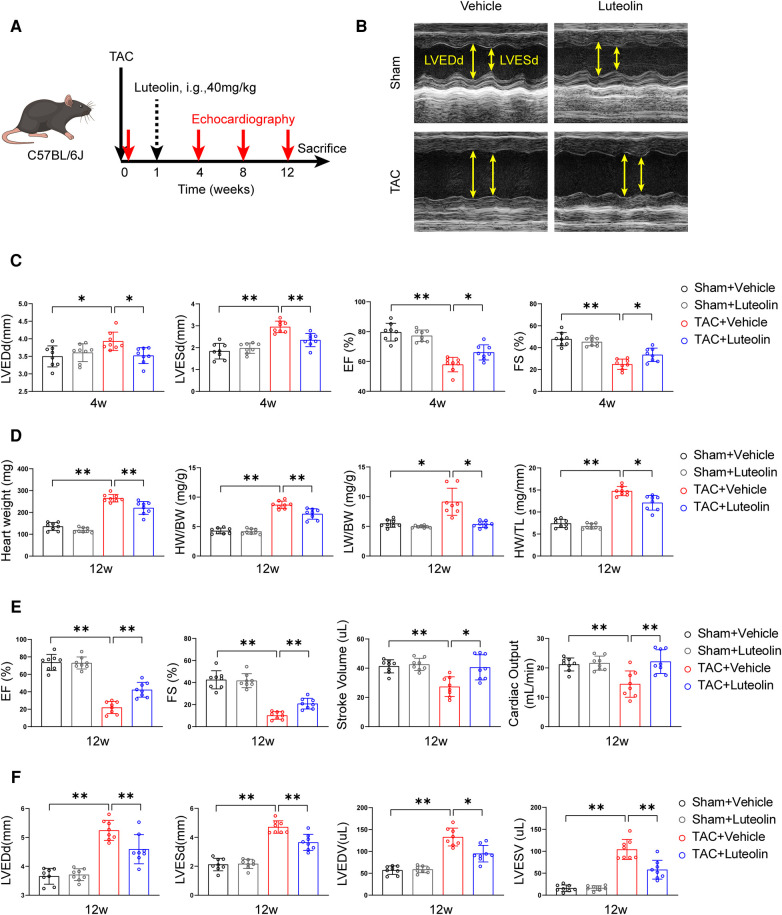
Luteolin inhibits cardiac dysfunction induced by pressure overload in mice. (**A**) Schematic diagram of the experimental procedure. (**B**) Representative echocardiography images of mice measured at 12 weeks after TAC. (**C**) Assessments of echocardiographic parameters of left ventricular end-diastolic diameter (LVEDd), left ventricular end-systolic diameter (LVESd), ejection fractions (EF), and fraction shortening (FS) of mice at 4 weeks after sham or TAC surgery (*n* = 8). (**D**) Heart weight (HW), HW/body weight (BW), lung weight (LW)/BW, and HW/tibia length (TL) ratios of mice at 12 weeks after sham or TAC surgery (*n* = 8). (**E,F**) Assessments of echocardiographic parameters of EF, FS, stroke volume, cardiac output, LVEDd, LVESd, left ventricular end-diastolic volume (LVEDV), and left ventricular end-systolic volume (LVESV) of mice at 12 weeks after sham or TAC surgery (*n* = 8). Values are presented as mean ± SD. *P < 0.05, **P < 0.01.

To evaluate whether luteolin ameliorated heart failure in mice, we randomly divided wild type mice (C57BL/6) into two groups for TAC or sham surgery. Each group were further randomly divided into two groups and orally administered with luteolin (40 mg/kg) or vehicle (1% CMC-Na) once daily at one week after surgery (Figure 3A). Cardiac function at 4, 8, and 12 weeks after TAC or sham surgery was detected by echocardiography, respectively (Supplementary Table S4). After 4 weeks of TAC, echocardiographic evaluation showed signs of cardiac hypertrophy and reduced cardiac function in TAC-treated mice compared to sham control, based on increased left ventricular end-diastolic diameter (LVEDd) and left ventricular end-systolic diameter (LVESd), reduced ejection fractions (EF) and fractional shortening (Figure 3C). Compared to the vehicle group, luteolin inhibited cardiac hypertrophy and deterioration of cardiac function in TAC mice (Figure 3C). After 12 weeks of TAC, mice were sacrificed for phenotypic and histological examination. Luteolin significantly blocked pressure overload-induced cardiac enlargement in TAC-treated mice (Figure 3D). Furthermore, echocardiographic evaluation, including EF, fractional shortening, stroke volume, cardiac output, LVEDd, LVESd, left ventricular end-diastolic volume, and left ventricular end-systolic volume, further verified the protective function of luteolin administration against cardiac remodeling and decreased cardiac function in TAC-treated mice after 12 weeks of TAC (Figures 3E,F). Notably, luteolin administration showed negligible influences in the sham settings (Figures 3B–F).

### Luteolin prevents cardiac hypertrophy and fibrosis in mice

**Figure 4:**
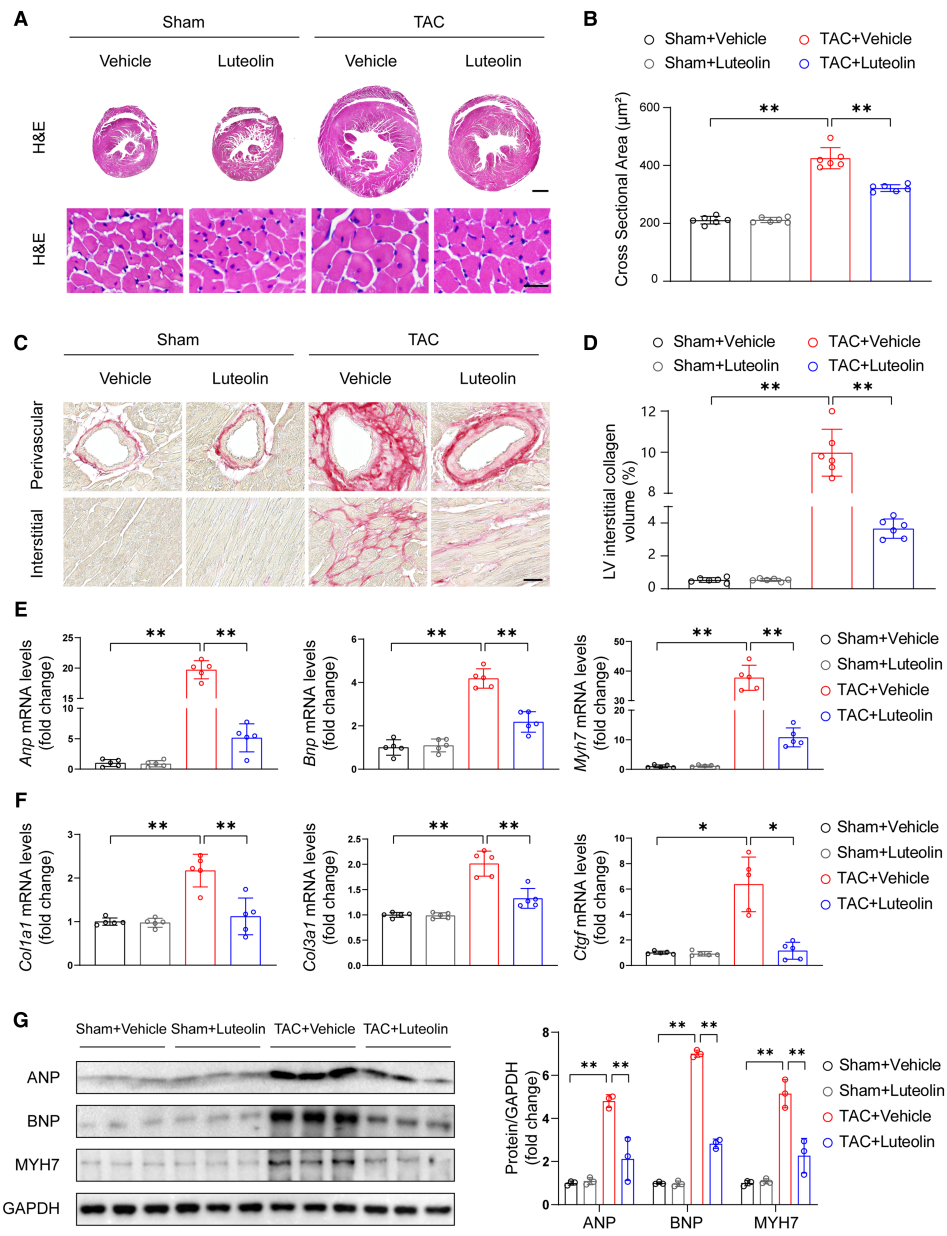
Luteolin prevents cardiac hypertrophy and fibrosis in mice. (**A**) Representative images of hematoxylin-eosin (H&E) staining of left ventricular cross-sections in the mice hearts at 12 weeks after sham or TAC surgery (*n* = 6). Scale bar, 1 mm for the top set and 25 μm for the bottom parts. (**B**) Quantitative results of average cross-sectional areas from the indicated groups. (**C**) Representative images of picrosirius red (PSR) staining of left ventricular cross-sections in the mice hearts at 12 weeks after sham or TAC surgery (*n* = 6). Scale bar, 50 μm. (**D**) Quantitative results of left ventricular interstitial collagen volume from the indicated groups. (**E,F**) Relative mRNA levels of hypertrophy and fibrosis marker genes in heart tissues from the indicated mice (*n* = 5). (**G**) Immunoblotting (left) and quantitation (right) of ANP, BNP, and MYH7 protein levels in the mice hearts at 12 weeks after sham or TAC surgery (*n* = 3). Values are presented as mean ± SD. *P < 0.05, **P < 0.01. ANP*,* atrial natriuretic peptide; BNP, b-type natriuretic peptide; PE, phenylephrine; MYH7, myosin heavy chain 7.

Sustained pathological overload induces maladaptation and cardiac remodeling, including cardiomyocyte hypertrophy, fibrosis, capillary rarefaction, cellular dysfunction, and other complex responses, ultimately leading to heart failure (21, 22). After 12 weeks of TAC, luteolin-treated TAC mice had significantly attenuated cross-sectional area of cardiomyocytes compared with TAC mice, as indicated by histological analysis with hematoxylin-eosin staining (Figures 4A,B). Furthermore, PSR staining of heart sections showed that luteolin treatment markedly inhibited cardiac fibrosis in TAC mice (Figures 4C,D). Consistent with these data, the expression levels of *Anp*, *Bnp*, *Myh7*, *Col3a1* (collagen type III alpha 1), *Col1a1* (collagen type I alpha 1), and *Ctgf* (connective tissue growth factor) were also notably downregulated in luteolin-treated TAC mice compared to the vehicle group (Figures 4E–G). These data clearly support that luteolin treatment attenuated the pathological cardiac remodeling induced by pressure overload.

### Luteolin enhances fatty acid metabolism and decreases glucose metabolism in the mouse failing hearts

**Figure 5:**
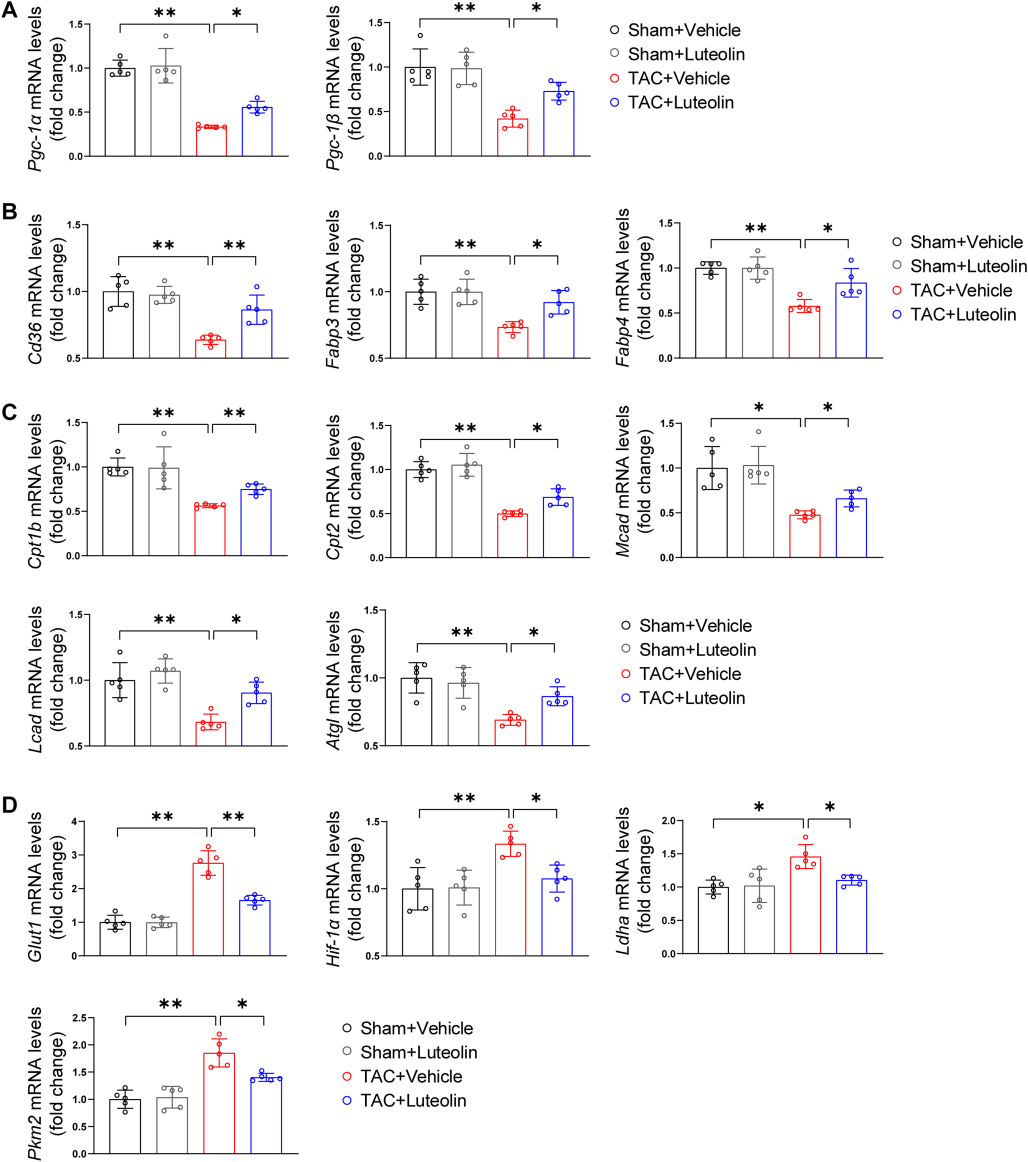
Luteolin enhances fatty acid metabolism and decreases glucose metabolism in the mouse failing hearts. (**A**) Relative mRNA levels of PPARγ coactivator-1α and PPARγ coactivator-1β in the mice hearts at 12 weeks after sham or TAC surgery (*n* = 5). (**B,C**) Relative mRNA levels of genes associated with fatty acid uptake (**B**) and fatty acid oxidation (**C**) in the mice hearts at 12 weeks after sham or TAC surgery (*n* = 5). (**D**) Relative mRNA levels of genes associated with glucose metabolism in the mice hearts at 12 weeks after sham or TAC surgery (*n* = 5). Values are presented as mean ± SD. *P < 0.05, **P < 0.01. *Pgc-1α/β*, peroxisome proliferative activated receptor-gamma coactivator-1*α*/*β*; *Fabp3/4*, fatty acid binding protein 3/4; *Cpt1b/2*, carnitine palmitoyltransferase 1b/2; *Mcad*, medium-chain acyl-CoA dehydrogenase; *Lcad*, long-chain acyl-CoA dehydrogenase; *Atgl*, adipose triglyceride lipase; *Glut1*, glucose transporter 1; *Hif-1α*, hypoxia-inducible factor 1α; PPARγ, peroxisome proliferator activated receptor γ; *Ldha*, lactate dehydrogenase A; *Pkm2*, pyruvate kinase M2.

It is known that in the case of pathological hypertrophy, the heart undergoes metabolism reprogramming characterized by an increased reliance on glucose metabolism and a reduced reliance on fatty acid oxidation. This metabolic profile reduces the capacity for ATP synthesis and ultimately promotes the progression of heart failure (23, 24). We detected the mRNA levels of genes associated with fatty acid and glucose metabolism in the mouse failing hearts. The mRNA levels of PPARγ coactivator-1α/1β, the critical regulators of fatty acid uptake and oxidation, were markedly reduced in TAC hearts, while luteolin attenuated this change (Figure 5A). Consistently, luteolin treatment reversed the reduction of mRNA levels of genes associated with fatty acid uptake (Figure 5B) and fatty acid oxidation (Figure 5C) in the heart from TAC-treated mice. The administration of luteolin treatment also reversed increased levels of genes associated with glucose metabolism (Figure 5D).

### Luteolin directly binds to and activates PPARγ during cardiac hypertrophy and HF

**Figure 6:**
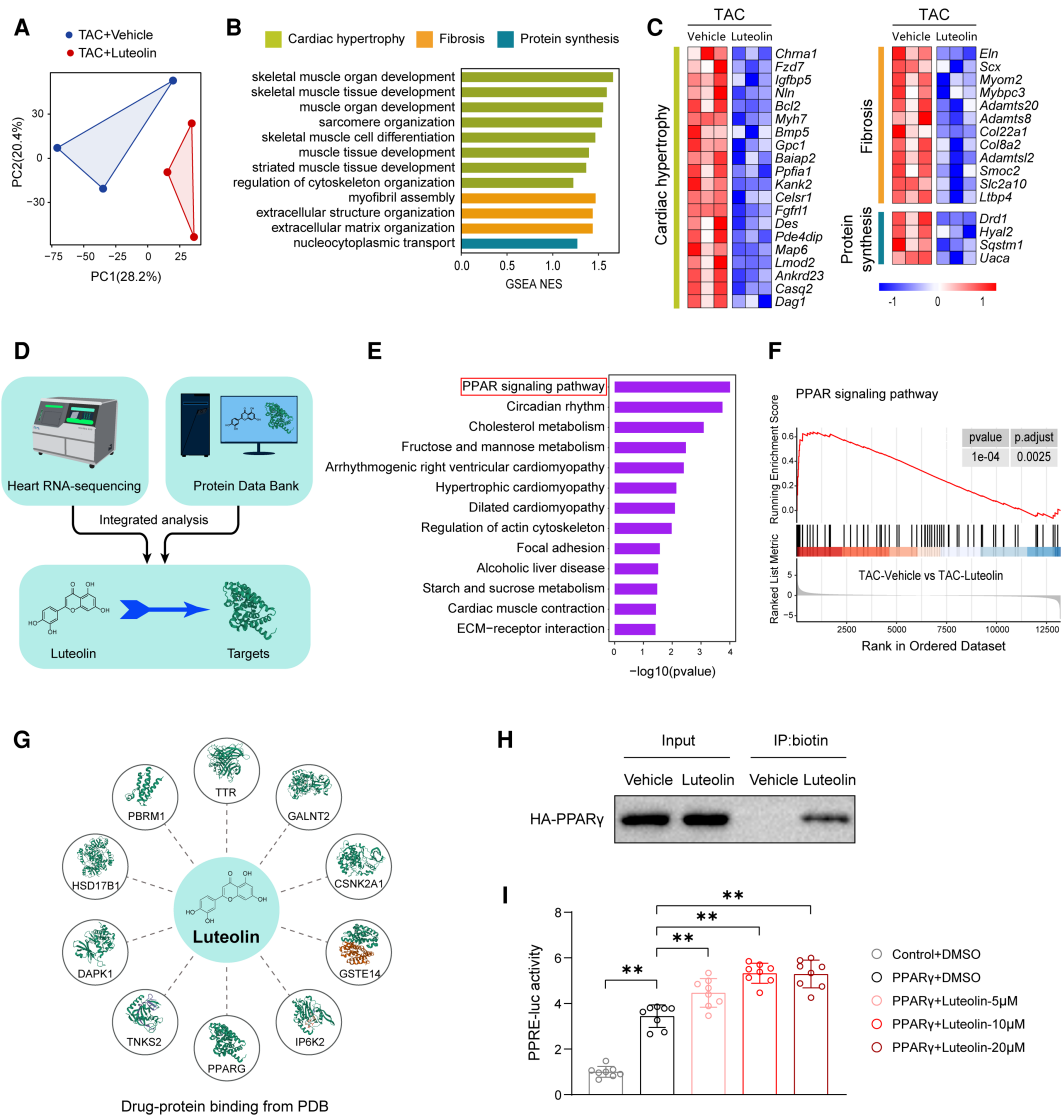
Luteolin directly binds to and activates PPARγ during cardiac hypertrophy and HF. (**A**) Principal component analysis showing the global sample distribution profiles between groups based on the RNA-sequencing data. (**B**) Gene set enrichment analysis of molecular events involved in cardiac hypertrophy, fibrosis, and protein synthesis in RNA-sequencing data. (**C**) Heatmap showing the significantly altered genes related to cardiac hypertrophy. (**D**) Schematic diagram of the conjoint analysis. (**E**) Kyoto Encyclopedia of Genes and Genomes pathway enrichment analysis of the identified differentially expressed genes. (**F**) Individual GSEA (gene set enrichment analysis) plots of PPAR signaling pathway. (**G**) Schematic diagram of luteolin-protein binding from protein data bank (PDB). (**H**) Biotinylated protein interaction pull-down assays showing the binding of luteolin and HA-tagged PPARγ protein in HEK 293 T cells. The data shown are representative of three independent experiments. (**I**) PPARγ-induced PPRE luciferase activity in the treatment of luteolin at three doses (5 μM, 10 μM, and 20 μM) in HEK 293 T cells. The data shown are representative of three independent experiments. Values are presented as mean ± SD. **P < 0.01. PPARγ, peroxisome proliferator activated receptor γ; PPRE, PPAR response element.

Considering the protective effects of luteolin on cardiac hypertrophy and HF, we tried to reveal the underlying molecular mechanisms of luteolin to ameliorates myocardial hypertrophy and heart failure. We analyzed RNA-sequencing data obtained from *in vivo* models. Principal component analysis revealed that the transcriptome profiles were clearly divided into two clusters (Figure 6A). GSEA analysis showed that the genes modulated by luteolin were mainly concentrated in cardiac hypertrophy, fibrosis, and protein synthesis (Figure 6B). The heatmap showed that luteolin markedly repressed the expression levels of genes associated with the aforesaid pathways (Figure 6C). To identify potential targets of luteolin, we performed a combined analysis of drug-target interacting investigations and RNA-sequencing (Figure 6D). First, Kyoto Encyclopedia of Genes and Genomes (KEGG) analysis demonstrated that PPAR signaling pathway was the most notably enriched molecular event regulated by luteolin (Figure 6E). Moreover, we performed gene set enrichment analysis, which further confirmed the upregulated PPAR signaling in the luteolin-treated group (Figure 6F). Then, we queried the protein data bank (PDB) and obtain 10 candidates interacting with luteolin (Figure 6G, Supplementary Table S5).

Combining the results of KEGG analysis with PDB data, we hypothesized that PPARγ may be a crucial target for luteolin in the inhibition of cardiac hypertrophy and HF. In order to determine whether luteolin could bind to PPARγ protein, we synthesized the chemical probe biotin-labeled luteolin (biotin-luteolin) and performed the biotinylated protein interaction pull-down assay. The data confirmed the interaction between luteolin and PPARγ (Figure 6H). Furthermore, we validated that luteolin dose-dependently activated the transcriptional activity of PPARγ in cultured HEK 293 T cells transfected with the PPAR response element (PPRE) reporters and PPARγ (Figure 6I). Thus, we speculated that PPARγ might be the direct target required for luteolin to exert a protective effect in cardiac hypertrophy and HF.

### Luteolin inhibits cardiac hypertrophy in a PPARγ-dependent manner

**Figure 7:**
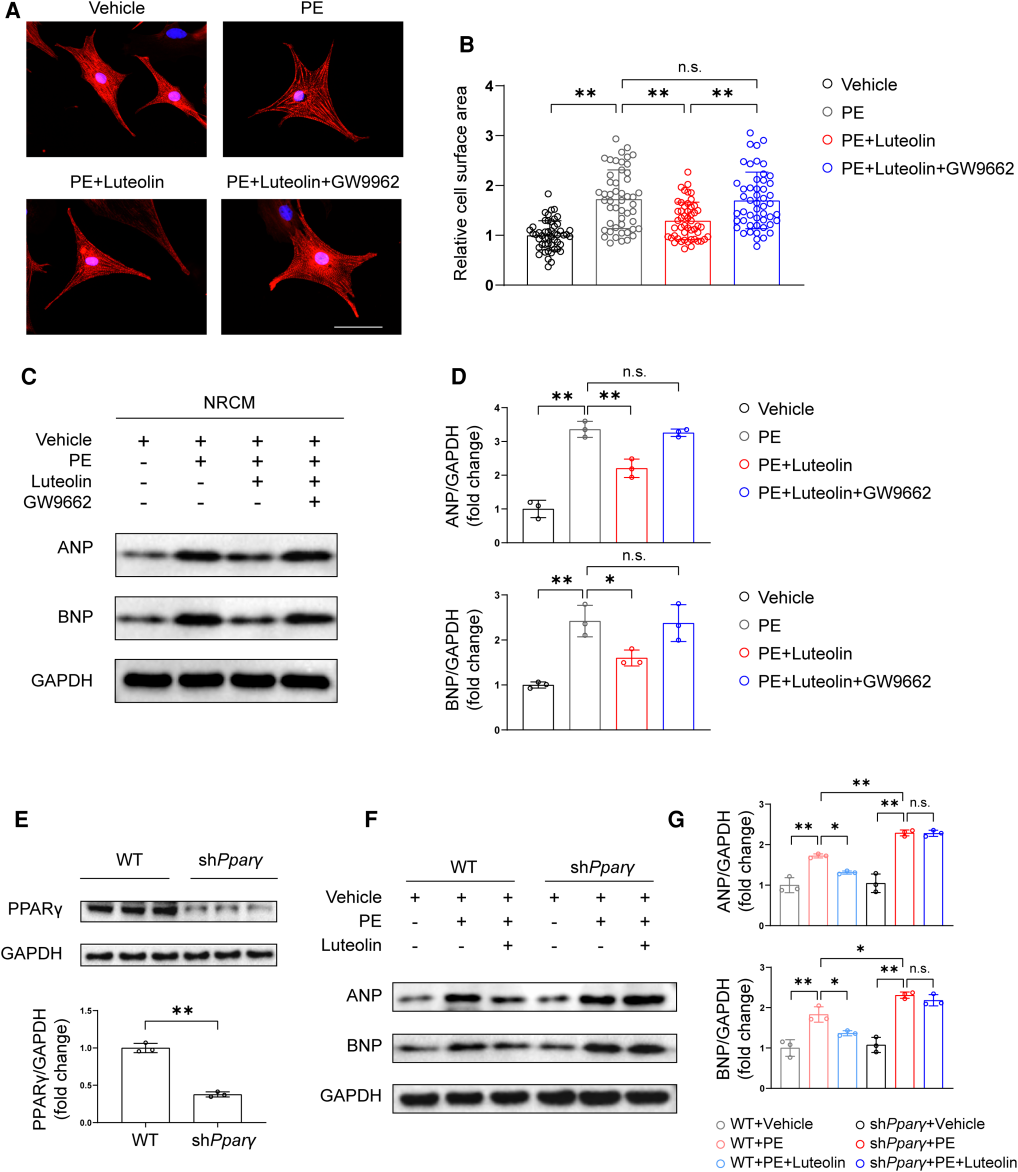
Luteolin inhibits cardiac hypertrophy in a PPARγ-dependent manner. (**A**) Representative immunofluorescence images of α-actinin staining of NRCMs treated with PBS, PE (50 μM), PE + luteolin (10 μM), or PE + luteolin + GW9662 (20 μM) for 24 h (*n* ≥ 50 cells per group). Scale bar, 50 μm. (**B**) Quantitative results of the cell surface area of NRCMs from the indicated groups. The data shown are representative of three independent experiments. (**C,D**) Immunoblotting analysis (**C**) and quantitative results (**D**) of ANP and BNP in cultured NRCMs treated with vehicle (PBS), PE (50 μM), PE + luteolin (10 μM), or PE + luteolin + GW9662 (20 μM) for 24 h. The data shown are representative of three independent experiments. (**E**) Immunoblotting analysis (top) and quantitative results (bottom) of PPARγ in cultured WT and PPARγ knockdown H9C2. (**F,G**) Immunoblotting analysis (**F**) and quantitative results (**G**) of ANP and BNP in cultured cultured WT and PPARγ knockdown H9C2 treated with vehicle (PBS), PE (50 μM), and PE + luteolin (10 μM) for 24 h. The data shown are representative of three independent experiments. Values are presented as mean ± SD. *P < 0.05, **P < 0.01, n.s., no significant difference. PPARγ, peroxisome proliferator activated receptor γ; NRCM, primary neonatal rat cardiomyocyte; ANP*,* atrial natriuretic peptide; BNP, b-type natriuretic peptide; PE, phenylephrine; WT, wild-type.

PPARγ is a critical modulator against cardiac hypertrophy (25–27), and its agonists have been found to inhibit cardiomyocytes hypertrophy by improving metabolic homeostasis and inflammatory response (28). To further evaluate whether PPARγ activation is required for the protective effect of luteolin, we cotreated NRCMs by luteolin in combined with a PPARγ activation inhibitor, GW9662. Remarkably, GW9662 treatment largely eliminated the effect of luteolin in ameliorating PE-induced cardiomyocyte hypertrophy, as shown by immunofluorescence staining and immunoblotting analysis (Figures 7A–D). Furthermore, we constructed *Pparγ* knockdown H9C2 cells (Figure 7E). Consistently, *Pparγ* knockdown abrogated the protective effect of luteolin on PE-induced expression of the relevant cardiac hypertrophy markers (Figures 7F,G).

### Luteolin elevates the stability of PPARγ *via* inhibiting PPARγ ubiquitination

**Figure 8:**
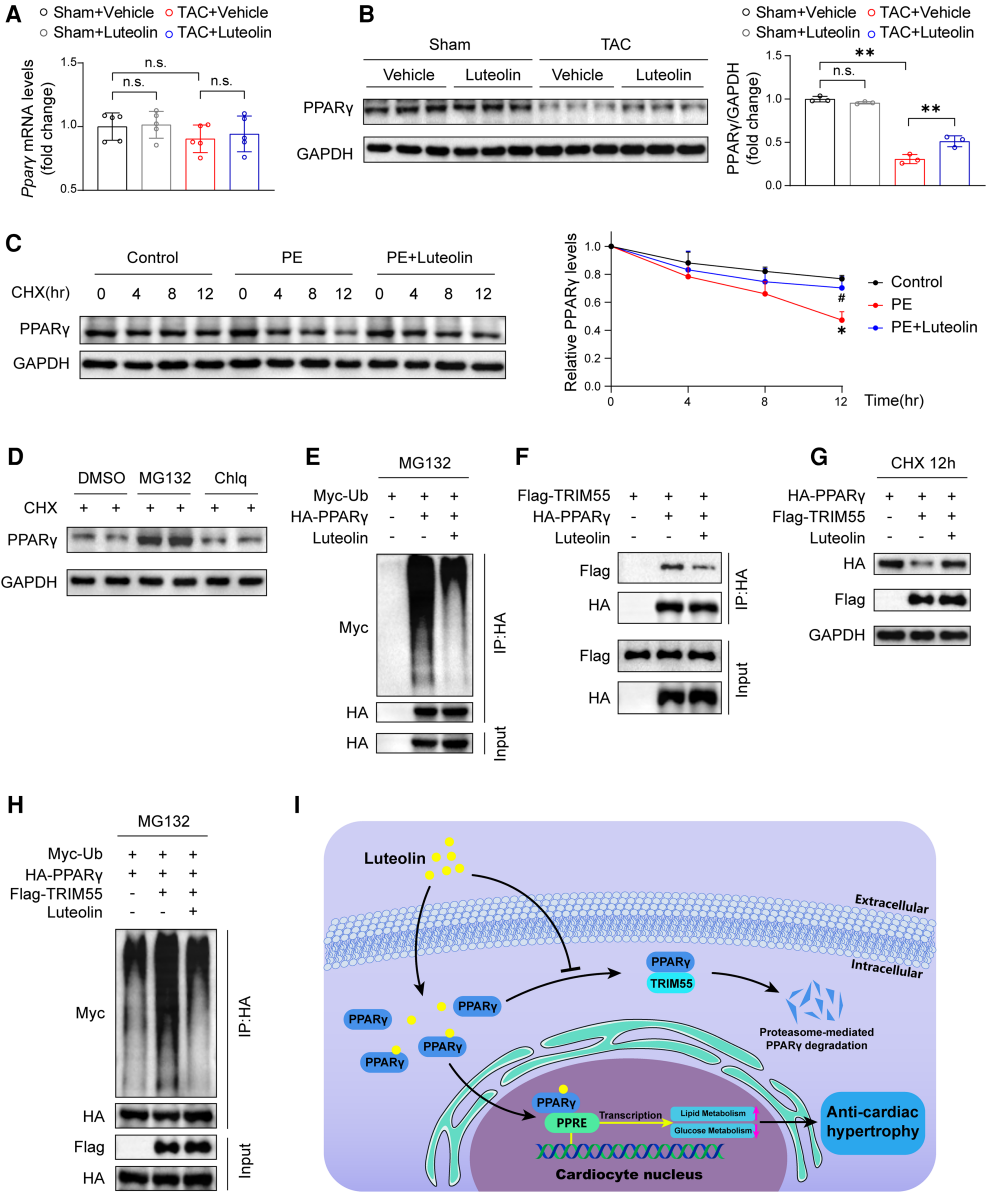
Luteolin elevates the stability of PPARγ *via* inhibiting PPARγ ubiquitination. (**A**) Relative mRNA levels of PPARγ in the mice hearts at 12 weeks after sham or TAC surgery (*n* = 5). Values are presented as mean ± SD. n.s., no significant difference. (**B**) Immunoblotting analysis (left) and quantitative results (right) of PPARγ in the mice hearts at 12 weeks after sham or TAC surgery (*n* = 5). Values are presented as mean ± SD. **P < 0.01, n.s., no significant difference. (**C**) Immunoblotting analysis (left) and quantitative results (right) of PPARγ protein in NRCMs exposed to CHX (100 μM) for the indicated time with or without the luteolin treatment. The data shown are representative of three independent experiments. Values are presented as mean ± SD. *P < 0.05 compared to the control group. ^#^*P *< 0.05 compared to the PE group. (**D**) PPARγ protein levels in NRCMs exposed to MG132 (10 μM) or Chlq (25 μM) in the presence of CHX (100 μM) for 4 h. The data shown are representative of three independent experiments. (**E**) The ubiquitination levels of PPARγ in HEK 293 T cells transfected with HA-tagged PPARγ and Myc-tagged Ub exposed to DMSO or luteolin (10 μM) in the presence of MG132 (10 μM). The data shown are representative of three independent experiments. (**F**) IP analyses of the interaction between TRIM55 and PPARγ in HEK 293 T cells transfected indicated plasmids exposed to DMSO or luteolin (10 μM). The data shown are representative of three independent experiments. (**G**) PPARγ protein levels in HEK 293 T cells transfected with HA-tagged PPARγ and Flag-tagged TRIM55 exposed to DMSO or luteolin (10 μM) in the presence of CHX (100 μM) for 12 h. The data shown are representative of three independent experiments. (**H**) The ubiquitination levels of PPARγ in HEK 293 T cells transfected with HA-tagged PPARγ, Flag-tagged TRIM55, and Myc-tagged Ub exposed to DMSO or luteolin (10 μM) in the presence of MG132 (10 μM). The data shown are representative of three independent experiments. (**I**) Schematic illustrating the model that luteolin is a promising therapeutic compound for pathological cardiac hypertrophy and heart failure by directly targeting PPARγ ubiquitin-proteasomal degradation and metabolic homeostasis. PPARγ, peroxisome proliferator activated receptor γ; DMSO, dimethyl sulfoxide; CHX, cycloheximide; Ub, ubiquitin; PE, phenylephrine; TRIM55, tripartite motif containing 55; NRCM, primary neonatal rat cardiomyocyte; PPRE, PPAR response element.

To explore the mechanism by which luteolin modulates PPARγ, we detected the mRNA and protein levels of PPARγ in heart samples from mice. Luteolin had no significant effect on PPARγ mRNA expression, whereas luteolin administration largely blocked TAC-induced decrease in PPARγ protein expression levels in mouse heart samples at 12 weeks after TAC surgery (Figures 8A,B). These results indicate that luteolin may activate PPARγ by regulating its protein stability. By treating NRCMs with cycloheximide (CHX), the half-life of PPARγ protein was remarkably extended in luteolin-treated NRCMs (Figure 8C). Furthermore, we found that the proteasome inhibitor MG132, rather than the lysosomal inhibitor chloroquine (Chlq), reversed CHX-induced destabilization of PPARγ, indicating that PPARγ was degraded mainly in a proteasome-dependent way (Figure 8D).

Subsequently, we investigated the impact of luteolin on PPARγ ubiquitination levels and found a dramatically reduced ubiquitination level of PPARγ by luteolin treatment (Figure 8E). A previous study reported that tripartite motif containing 55 (TRIM55), a muscle-specific ubiquitin ligase, mediates the ubiquitination-mediated degradation of PPARγ in myocardial tissue (29). We first confirmed the interaction with and enhancing ubiquitination function of TRIM55 on PPARγ (Figures 8F–H), and further examined the impact of luteolin on PPARγ and TRIM55 complex. The data revealed that luteolin weakened the interaction between TRIM55 and PPARγ and largely reversed TRIM55-induced enhancement of PPARγ ubiquitination level (Figures 8F–H). In summary, PPARγ is the direct pharmacological target of luteolin, and luteolin stabilizes PPARγ protein expression by inhibiting TRIM55-mediated ubiquitinational degradation (Figure 8I).

## Discussion

In this study, based on a high-throughput FDA drug screening, we identified luteolin as a candidate for the management of cardiac hypertrophy. Further *in vitro* and *in vivo* experiments verified that luteolin can significantly attenuate pathological cardiac hypertrophy and HF mainly by activating PPARγ pathway. Mechanistically, luteolin treatment stabilizes PPARγ by inhibiting its ubiquitination, thereby indirectly regulating fatty acid and glucose metabolism to exert a protective effect on the heart.

Luteolin is one of the most prevalent flavones flavonoids and is abundant in a wide range of vegetables, fruits, and herbs (10). Although the therapeutic effects of luteolin on cardiac hypertrophy and HF have been sporadic suggested (11, 13, 14, 30), systemic studies in this regard are still missing. Meanwhile the detailed molecular events and gene expression profiles associated with the histological phenotypes induced by luteolin treatment are also not clear. All these information is imperative for developing luteolin for the treatment against cardiac hypertrophy. To date, previous studies have repeatedly attributed the therapeutic effects of luteolin to suppressing oxidative stress, inflammatory responses, autophagy, and apoptosis *via* remaining to be verified molecular target (10). In this study, beyond phenotypic verification, we systematically illustrated that luteolin treatment universally downregulated genes associated with cardiac hypertrophy, fibrosis, and protein synthesis by RNA-sequencing. Notably, we first verified that luteolin can directly bind to PPARγ, a crucial regulator of metabolic homeostasis, prevents its ubiquitination mediated degradation, thereby exerts the protective effect against cardiac hypertrophy and heart failure. And this mechanism is novel relative to what we know regarding the protective effect of luteolin.

PPARγ is a nuclear receptor that regulates glucose and fatty acid metabolism (31). PPARγ promotes lipid droplet formation and triglyceride lipolysis in myocardial tissue, thus inhibiting the accumulation of cardiac cytotoxic lipids (24). Cardiomyocyte-specific PPARγ deficiency induces cardiac hypertrophy in mice (25), whereas overexpression of PPARγ in cardiomyocyte enhances cardiac uptake of lipids and glucose (32). Although the therapeutic outcomes of transgenic mice and rosiglitazone are ambiguous (25, 32), there is increasing evidence that PPARγ is a protective modulator in cardiac hypertrophy (26–28, 33) and heart failure (31, 34). PPARγ and the downstream signaling can be activated by promoting its expression, stimulating its activity, and preventing its degradation. While various PPARγ agonists have been developed, their application has been limited by hepatotoxicity, cancer risk and cardiac side effects (35). The expression booster and protein level stabilizer have not been sufficiently explored. In this study, following finding luteolin prevents cardiac hypertrophy and heart failure *via* activating PPARγ pathway, and we also found that by binding to PPARγ luteolin interrupted the interaction between TRIM55 and PPARγ and prevents the TRIM55-mediated proteasome-dependent degradation of PPARγ. Notably, the role of PPARγ in cancer progression is still in debate. Although there is substantial evidence that PPARγ acts as a tumor suppressor and inhibits tumor cell growth in a variety of cancers, its pro-tumor potential should not be overlooked (36, 37). Considering the complex role of PPARγ in metabolic regulation and cancer progression, selective PPARγ modulators for cell- or organ-specific modulation are a promising area for future studies. While the PPARγ agonists haven't been able to be applied in clinic, our study demonstrated that the strategies of stabilizing PPARγ can be a key alternative way for pathological cardiac hypertrophy and HF treatment.

It is well known that cardiac metabolism disturbances including the relative lack of energy production and the altered source of energy substrates are thought to be associated with impaired cardiac function in failing failure (38). During heart failure induced by the prolonged presence of pressure load, there are two significant adaptive changes in myocardial energy metabolism (24, 39). In the first place, an enlargement of heart weight is accompanied by increased myocardial energy consumption. Secondly, pressure load and low cardiac output chronically activate the renin-angiotensin-aldosterone system and the sympathetic nervous system, altering the nutrient supply to the heart by activating gluconeogenesis, ketogenesis, and lipolysis (40). Overall, the failing heart is more inclined to glucose (41), ketone bodies, and lactate (42) as energy suppliers, while the proportion of energy supply from fatty acid uptake and oxidation is reduced (43). In addition, the low oxidative phosphorylation capacity leads to low cardiac metabolic reserve, decrease cardiomyocyte high-energy phosphate content, and ultimately leads to poorer cardiac contractility (44). Considering PPARγ as a crucial regulator of glucose and fatty acid metabolism, we detected the mRNA levels of genes associated with glucose and fatty acid metabolism in the mice hearts. Interestingly, luteolin effectively reversed cardiac hypertrophy and subsequent HF by augmenting fatty acid metabolism and inhibiting glucose metabolism in failing heart, which may endow luteolin more potential for pathological cardiac hypertrophy and heart failure treatment relative to known therapeutics.

## Conclusion

In summary, our results first revealed that luteolin prevents cardiac hypertrophy and HF by regulating myocardial fatty acid and glucose metabolism, which relies on PPARγ activation. luteolin binds to PPARγ and then interferes with the interaction between PPARγ and TRIM55, reduces the ubiquitination of PPARγ induced by TRIM55, stabilising the PPAR γ protein, and ultimately improves myocardial fatty acid and glucose metabolism. The above results offer new insights into the pathogenesis of pathological cardiac hypertrophy and HF, and add novel evidence for the benefits of PPARγ activation in cardiac hypertrophy and HF.

## Data Availability

The datasets presented in this study can be found in online repositories. The names of the repository/repositories and accession number(s) can be found at: https://www.ncbi.nlm.nih.gov/, PRJNA939393; https://www.ncbi.nlm.nih.gov/, PRJNA939371.
