# Supplementary material for: A high-throughput drug screening identifies luteolin as a therapeutic candidate for pathological cardiac hypertrophy and heart failure
Source: Front Cardiovasc Med. 2023 Mar 14;10:1130635. doi: 10.3389/fcvm.2023.1130635 (PMC10043402; doi:10.3389/fcvm.2023.1130635)
Supplement: Supplementary file 1 [file Table1.docx]

**Supplementary Tables**

**Supplementary Table 1. Primer sequences for molecular cloning.**

| **Gene** | **Forward primer (5’-3’)** | **Reverse primer (5’-3’)** |
| --- | --- | --- |
| Rat *Bnp* promoter (-2147, +132 bp) | CTTTGGCGCCGGCTCGAGTCAAGGGCAGCCTAATCTA | ATATACCCTCTAGGTACCGGCAGCACCTTCTGGAGATCC |
| Rat *Myh7* promoter (-2500, +89 bp) | CTTTGGCGCCGGCTCGAGGGGCTCTAAAGTAGGAAAGT | ATATACCCTCTAGGTACCTCTGCGCCTCCAGCCGCT |
| Rat *Pparγ* shRNA | CCGGGAAGCTGTGAACCACTAATATCTCGAGATATTAGTGGTTCACAGCTTCTTTTTG | AATTCAAAAAGAAGCTGTGAACCACTAATATCTCGAGATATTAGTGGTTCACAGCTTC |

**Supplementary Table 2. Antibodies used in this study.**

| **Antibody** | **Manufacturer** | **Catalog number** | **Source of species** |
| --- | --- | --- | --- |
| ANP | Santa Cruz | sc-20158 | Rabbit |
| BNP | ABclonal | A2179 | Rabbit |
| MYH7 | Proteintech | 22280-1-AP | Rabbit |
| PPARγ | Cell Signaling Technology | 2435 | Rabbit |
| HA | Medical & Biological Laboratories | M180-3 | Mouse |
| Flag | Medical & Biological Laboratories | M185-3L | Mouse |
| Myc | Medical & Biological Laboratories | M047-3 | Mouse |
| GAPDH | Medical & Biological Laboratories | M171-3 | Mouse |

**Supplementary Table 3. Primer sequences for qPCR.**

| **Gene** | **Species** | **Forward primer (5’-3’)** | **Reverse primer (5’-3’)** |
| --- | --- | --- | --- |
| *Anp* | Mouse | TCGGAGCCTACGAAGATCCA | TTCGGTACCGGAAGCTGTTG |
| *Bnp* | Mouse | GAAGGACCAAGGCCTCACAA | TTCAGTGCGTTACAGCCCAA |
| *Myh7* | Mouse | CAACCTGTCCAAGTTCCGCA | TACTCCTCATTCAGGCCCTTG |
| *Col1a1* | Mouse | TGCTAACGTGGTTCGTGACCGT | ACATCTTGAGGTCGCGGCATGT |
| *Col3a1* | Mouse | ACGTAAGCACTGGTGGACAG | CCGGCTGGAAAGAAGTCTGA |
| *Ctgf* | Mouse | TGACCCCTGCGACCCACA | TACACCGACCCACCGAAGACACAG |
| *Gapdh* | Mouse | ATGTGTCCGTCGTGGATCTG | AGTTGGGATAGGGCCTCTCTT |
| *Anp* | Rat | AAAGCAAACTGAGGGCTCTGCTCG | TTCGGTACCGGAAGCTGTTGCA |
| *Bnp* | Rat | TGCCCCAGATGATTCTGCTC | TGTAGGGCCTTGGTCCTTTG |
| *Myh7* | Rat | AGTTCGGGCGAGTCAAAGATG | CAGGTTGTCTTGTTCCGCCT |
| *Gapdh* | Rat | ACTCTACCCACGGCAAGTTC | TGGGTTTCCCGTTGATGACC |
| *Pparγ* | Mouse | CAGTGATATCGACCAGCTGAA | CATGAATCCTTGGCCCTCT |
| *Cd36 (Fat)* | Mouse | TGCTGGAGCTGTTATTGGTG | TGGGTTTTGCACATCAAAGA |
| *Fabp3* | Mouse | CATCGAGAAGAACGGGGATA | TGCCATGAGTGAGAGTCAGG |
| *Fabp4* | Mouse | AAGAAGTGGGAGTGGGCTTT | TCGACTTTCCATCCCACTTC |
| *Cpt1b* | Mouse | GTCGCTTCTTCAAGGTCTGG | AAGAAAGCAGCACGTTCGAT |
| *Cpt2* | Mouse | TCCTCGATCAAGATGGGAAC | GATCCTTCATCGGGAAGTCA |
| *Acadm(Mcad)* | Mouse | GGCAAATGCCTGTGATTCTT | ACCCATTGCGATCTTGAAAC |
| *Acadl(Lcad)* | Mouse | ACTTGGGAAGAGCAAGCGTA | TTCCGTTTTCCACCAAAAAG |
| *Atgl* | Mouse | ACAGTGTCCCCATTCTCAGG | TTGGTTCAGTAGGCCATTCC |
| *Glut1* | Mouse | GCTGTGCTTATGGGCTTCTC | AGAGGCCACAAGTCTGCATT |
| *Hif-1α* | Mouse | TCACCAGACAGAGCAGGAAA | CTTGAAAAAGGGAGCCATCA |
| *Pgc-1α* | Mouse | GTAAATCTGCGGGATGATGG | AGCAGGGTCAAAATCGTCTG |
| *Pgc-1β* | Mouse | TGAGGTGTTCGGTGAGATTG | CCATAGCTCAGGTGGAAGGA |
| *Ldha* | Mouse | CAAAGACTACTGTGTAACTGCGA | TGGACTGTACTTGACAATGTTGG |
| *Pkm2* | Mouse | GTCTGGAGAAACAGCCAAGG | CGGAGTTCCTCGAATAGCTG |

**Supplementary Table 4. Echocardiographic analysis after Sham or TAC surgery for 8 weeks.**

| **Parameter** | **Sham+Vehicle(n=8)** | **Sham+Luteolin(n=8)** | **TAC+Vehicle(n=8)** | **TAC+Luteolin(n=8)** |
| --- | --- | --- | --- | --- |
| HR(bpm) | 518.81±16.33 | 504.23±6.92 | 504.60±15.25 | 495.55±34.89 |
| LVEDd(mm) | 3.80±0.20 | 3.49±0.32 | 4.60±0.50** | 3.93±0.32**##** |
| LVESd(mm) | 2.27±0.24 | 1.91±0.27 | 3.79±0.60** | 2.70±0.82**##** |
| EF(%) | 71.09±7.19 | 76.84±6.57 | 36.91±10.45** | 55.07±9.01**##** |
| FS(%) | 40.18±5.92 | 45.08±6.70 | 17.89±5.70** | 28.46±5.82**#** |
| LV mass(mg) | 123.90±13.19 | 127.81±10.31 | 271.35±25.58** | 222.96±28.20**#** |

***P*<0.01 compared to the Sham-Vehicle group. #*P*<0.05 and ##*P*<0.01 compared to the TAC-Vehicle group. All values are presented as means ± SD. HR, heart rate; LVEDd, left ventricular end-diastolic diameter; LVESd, left ventricular end-systolic diameter; EF, ejection fractions; FS, fraction shortening.

**Supplementary Table 5.** **Summary of proteins interacting with luteolin obtained by querying the protein data bank.**

| **Study** | **Protein Name** | **Effects of Luteolin** |
| --- | --- | --- |
| 26020516, Iakovleva I | Transthyretin (TTR) | prevents TTR mediated toxic response |
| 29061849, Liu F | polypeptide N-acetyl-α-galactosaminyltransferase 2 (GALNT2) | inhibits the activity of GALNT2 |
| 22794353, Lolli G | Casein kinase II subunit alpha (CSNK2A1) | inhibits the activity of casein kinase II |
| 22391103, Puhl AC | Peroxisome Proliferator Activated Receptor Gamma (PPARG) | a partial PPARγ agonist |
| 30624931, Gu C | Inositol Hexakisphosphate Kinase 2 (IP6K2) | inhibits the activity of IP6K2 |
| 35172816, Inaba K | Glutathione S-transferase Noppera-bo (GSTE14) | inhibits the activity of GSTE14 |
| 23574272, Narwal M | Tankyrase 2 (TNKS2) | inhibits the activity of TNKS2 |
| 26322379, Yokoyama T | Death Associated Protein Kinase 1 (DAPK1) | inhibits the activity of DAPK1 |
| 28259640, Cassetta A | 17-beta-Hydroxysteroid Dehydrogenase Type 1 (HSD17B1) | inhibits the activity of 17β-hydroxysteroid dehydrogenases |
| 27617704, Myrianthopoulos V | Polybromo 1 (PBRM1) | / |
